# Supplementary material for: G9a Knockdown Suppresses Cancer Aggressiveness by Facilitating Smad Protein Phosphorylation through Increasing BMP5 Expression in Luminal A Type Breast Cancer
Source: Int J Mol Sci. 2022 Jan 6;23(2):589. doi: 10.3390/ijms23020589 (PMC8776044; doi:10.3390/ijms23020589)
Supplement: Supplementary file 1 [file ijms-23-00589-s001.zip › ijms-1513099-supplementary.pdf]

Table S1. The patients with stage 3 disease showed higher BMP5 levels than those of Stage 2.

No.	Age	Pathology diagnosis (BR1507)	TNM	Grade	Stage	BMP5
A1	50	Invasive ductal carcinoma	T2N0M0	1	IIA	2
A2						2
A3						2
A4	45	Invasive ductal carcinoma	T3N0N0	2	IIB	2
A5						2
A6						2
A7	54	Invasive ductal carcinoma	T2N0M0	3	IIA	3
A8						2
A9						3
A10	50	Invasive ductal carcinoma	T3N0M0	1	IIB	2
A11						2
A12						1
A13	71	Invasive ductal carcinoma	T4N2M0	2	IIIB	2
A14						1
A15						3
B1	52	Invasive ductal carcinoma	T3N1M0	2	IIIA	0
B2						0
B3						1
B4	70	Invasive ductal carcinoma	T4N2M0	1	IIIB	2
B5						1
B6						1
B7	55	Invasive ductal carcinoma	T2N1M0	2	IIB	2
B8						1
B9						1
B10	48	Invasive ductal carcinoma	T2N1M0	1	IIB	2
B11						2
B12						2
B13	43	Invasive ductal carcinoma	T2N1M0	1	IIB	3
B14						3
B15						3
C1	52	Invasive ductal carcinoma	T2N0M0	2	IIA	1
C2						1
C3						1
C4	23	Invasive ductal carcinoma	T3N1M0	2	IIIA	1
C5						2
C6						2
C7	61	Invasive ductal carcinoma	T2N1M0	2	IIB	1
C8						2
C9						1
C10	51	Invasive ductal carcinoma	T3N2M0	2	IIIA	2
C11						2
C12						3
C13	68	Invasive ductal carcinoma	T3N0M0	2	IIB	3
C14						3
C15						3
D1	51	Invasive ductal carcinoma	T2N0M0	2	IIA	2
D2						1
D3						2
D4	52	Invasive ductal carcinoma	T2N0M0	3	IIA	3
D5						n.d.
D6						3
D7	74	Invasive ductal carcinoma				2

D8			T2N1M0	1	IIB	2
D9						2
D10						2
D11	56	Invasive ductal carcinoma	T2N0M0	2	IIA	2
D12						2
D13						2
D14	49	Invasive ductal carcinoma	T2N0M0	1	IIA	2
D15						2
E1						1
E2	53	Invasive ductal carcinoma	T4N0M0	2	IIIB	1
E3						0
E4						1
E5	37	Invasive ductal carcinoma	T4N0M0	1	IIIB	1
E6						2
E7						1
E8	57	Invasive ductal carcinoma	T2N0M0	2	IIA	2
E9						3
E10						2
E11	48	Invasive ductal carcinoma	T2N1M0	2	IIB	3
E12						2
E13						1
E14	58	Invasive ductal carcinoma	T3N1M0	2	IIIA	1
E15						1
F1						1
F2	43	Invasive ductal carcinoma	T2N0M0	1	IIA	2
F3						3
F4						2
F5	62	Invasive ductal carcinoma	T2N0M0	2	IIA	2
F6						1
F7						3
F8	61	Invasive ductal carcinoma	T2N0M0	2	IIIB	3
F9						3
F10						3
F11	55	Invasive ductal carcinoma	T2N0M0	1	IIA	2
F12						2
F13						1
F14	63	Invasive ductal carcinoma	T4N0M0	2	IIIB	1
F15						1
G1						1
G2	42	Invasive ductal carcinoma	T2N1M0	2	IIB	1
G3						2
G4						0
G5	55	Invasive ductal carcinoma	T2N2M0	2	IIIA	0
G6						1
G7						2
G8	81	Invasive ductal carcinoma	T2N2M0	2	IIIA	2
G9						2
G10						3
G11	78	Invasive ductal carcinoma	T4N0M0	2	IIIB	3
G12						3
G13						2
G14	37	Invasive ductal carcinoma	T2N2M0	2	IIIA	3
G15						2
H1	34	Invasive ductal carcinoma				0

H2			T4N0M0	2	IIIB	0
H3						1
H4	69	Invasive ductal carcinoma	T3N0M0	2	IIB	0
H5						0
H6						0
H7	41	Invasive ductal carcinoma	T2N0M0	1	IIA	2
H8						2
H9						2
H10	47	Invasive ductal carcinoma	T4N1M0	2	IIIB	2
H11						2
H12						2
H13	56	Invasive ductal carcinoma	T2N0M0	2	IIA	0
H14						1
H15						2
I1	50	Invasive ductal carcinoma	T2N1M0	2	IIB	1
I2						1
I3						1
I4	40	Invasive ductal carcinoma	T2N0M0	2	IIA	1
I5						2
I6						1
I7	38	Invasive lobular carcinoma	T2N0M0	-	IIA	1
I8						2
I9						2
I10	49	Invasive lobular carcinoma	T2N0M0	-	IIA	3
I11						2
I12						3
I13	49	Invasive ductal carcinoma	T2N0M0	2	IIA	3
I14						3
I15						2
J1	43	Invasive papillary carcinoma	T2N1M0	-	IIB	1
J2						1
J3						1
J4	40	Invasive ductal carcinoma	T3N0M0	2	IIB	0
J5						0
J6						0
J7	45	Invasive ductal carcinoma	T2N1M0	2	IIB	1
J8						1
J9						2
J10	35	Invasive ductal carcinoma	T3N0M0	2	IIB	1
J11						1
J12						1
J13	50	Invasive ductal carcinoma	T4N2M0	2	IIIB	2
J14						2
J15						2

No.	Age	Pathology diagnosis (BR1508)	TNM	Grade	Stage	BMP5
A1	42	Invasive ductal carcinoma	T3N0N0	2	IIB	3
A2						3
A3						2
A4	70	Invasive ductal carcinoma	T3N0N0	2	IIB	1
A5						1
A6						2
A7	48	Invasive ductal carcinoma	T2N0M0	3	IIA	3
A8						3

A9						3
A10						1
A11	46	Invasive ductal carcinoma	T3N0M0	2	IIB	2
A12						2
A13						3
A14	46	Invasive ductal carcinoma	T2N0M0	2	IIA	2
A15						3
B1						3
B2	54	Invasive ductal carcinoma	T3N0N0	2	IIB	3
B3						3
B4						2
B5	36	Invasive ductal carcinoma	T3N0N0	1	IIB	2
B6						2
B7						2
B8	52	Invasive ductal carcinoma	T3N0N0	2	IIB	3
B9						3
B10						2
B11	31	Invasive ductal carcinoma	T4N2M0	1	IIIB	2
B12						3
B13						3
B14	48	Invasive ductal carcinoma	T2N0M0	1	IIA	3
B15						3
C1						2
C2	48	Invasive ductal carcinoma	T4N1M0	2	IIIB	2
C3						2
C4						3
C5	37	Invasive ductal carcinoma	T2N0M0	3	IIA	3
C6						2
C7						2
C8	60	Invasive ductal carcinoma	T3N0M0	2	IIB	3
C9						2
C10						2
C11	75	Invasive ductal carcinoma	T2N1M0	2	IIB	2
C12						3
C13						3
C14	58	Invasive ductal carcinoma	T3N2M0	2	IIIA	3
C15						3
D1						3
D2	50	Invasive ductal carcinoma	T4N1M0	3	IIIB	2
D3						2
D4						1
D5	28	Invasive ductal carcinoma	T2N1M0	2	IIB	2
D6						1
D7						1
D8	40	Invasive ductal carcinoma	T2N0M0	2	IIA	1
D9						2
D10						3
D11	36	Invasive ductal carcinoma	T2N0M0	3	IIA	3
D12						3
D13						3
D14	46	Invasive ductal carcinoma	T3N0M0	2	IIB	3
D15						3
E1	42	Invasive ductal carcinoma				1
E2			T4N0M0	2	IIIB	1

E3						1
E4	42	Invasive ductal carcinoma	T2N1M0	1-2	IIB	1
E5						2
E6						2
E7	36	Invasive ductal carcinoma	T3N0M0	1	IIB	3
E8						3
E9						3
E10	66	Invasive ductal carcinoma	T2N0M0	2	IIA	2
E11						3
E12						3
E13	49	Invasive ductal carcinoma	T4N0M0	2	IIIB	2
E14						1
E15						2
F1	49	Invasive ductal carcinoma	T2N0M0	2	IIA	1
F2						1
F3						0
F4	48	Invasive ductal carcinoma	T2N2M0	3	IIIA	2
F5						1
F6						2
F7	37	Invasive ductal carcinoma	T3N0M0	3	IIB	2
F8						3
F9						2
F10	48	Invasive ductal carcinoma	T3N0M0	3	IIB	1
F11						2
F12						1
F13	42	Invasive ductal carcinoma	T2N0M0	2	IIA	2
F14						3
F15						2
G1	42	Invasive ductal carcinoma	T4N2M0	1	IIIB	2
G2						1
G3						1
G4	50	Invasive ductal carcinoma	T2N2M0	2	IIIA	2
G5						1
G6						3
G7	63	Invasive ductal carcinoma	T2N0M0	3	IIA	2
G8						2
G9						2
G10	34	Invasive ductal carcinoma	T2N0M0	2	IIA	3
G11						2
G12						3
G13	50	Invasive ductal carcinoma	T2N0M0	3	IIA	3
G14						3
G15						3
H1	39	Invasive ductal carcinoma	T2N1M0	2	IIB	2
H2						1
H3						2
H4	54	Invasive ductal carcinoma	T4N0M0	3	IIIB	0
H5						0
H6						0
H7	49	Invasive ductal carcinoma	T2N0M0	2	IIA	2
H8						2
H9						3
H10	55	Invasive ductal carcinoma	T4N2M0	2	IIIB	0
H11						0

H12						0
H13	32	Invasive ductal carcinoma	T3N1M0	2	IIIA	1
H14						1
H15						1
I1	53	Invasive ductal carcinoma	T4N1M0	2-3	IIIB	0
I2						0
I3						0
I4	44	Invasive ductal carcinoma	T2N0M0	3	IIA	1
I5						1
I6						1
I7	70	Invasive ductal carcinoma	T4N2M0	2	IIIB	1
I8						1
I9						1
I10	48	Invasive ductal carcinoma	T2N1M0	2	IIB	1
I11						1
I12						1
I13	68	Invasive ductal carcinoma	T2N1M0	2	IIB	2
I14						2
I15						2
J1	28	Invasive cribriform carcinoma	T3N0M0	2	IIB	0
J2						1
J3						1
J4	48	Squamous cell carcinoma	T4N0M0	2	IIIB	0
J5						0
J6						0
J7	42	Mixed invasive lobular and duct carcinoma	T2N1M0	-	IIB	1
J8						1
J9						1
J10	48	Medullary carcinoma	T3N0M0	-	IIB	1
J11						1
J12						1
J13	41	Medullary carcinoma	T2N1M0	-	IIB	3
J14						3
J15						3

n.d. not determined

Scoring	0	1	2	3	4	5
% of stained cells	<10%	10~20%	20~40%	40~60%	60~80%	80~100%
